# Anatomical features of aneurysm sac, thrombus volume and localization and their impact on post-EVAR outcomes

**DOI:** 10.3389/fsurg.2026.1760159

**Published:** 2026-05-19

**Authors:** Emre Kulahcioglu, Mehmet Ali Türkcü, Fatma Akca, Bekir Boğaçhan Akkaya, Bahadır Aytekin, Hakkı Zafer İşcan

**Affiliations:** 1 Department of Cardiovascular Surgery, TC Saglik Bakanligi Kilis Prof Dr Alaeddin Yavasca Devlet Hastanesi, Kilis, Türkiye; 2 Department of Cardiovascular Surgery, TC Saglik Bakanligi Iskenderun Devlet Hastanesi, Iskenderun, Türkiye; 3 Department of Cardiovascular Surgery, TC Saglik Bakanligi Kirikkale Yuksek Ihtisas Hastanesi, Kirikkale, Türkiye; 4 Department of Cardiovascular Surgery, TC Saglik Bakanligi Ankara Sehir Hastanesi, Çankaya, Türkiye

**Keywords:** EVAR, inferior mesenteric artery, lumbar arteries, sac volume shrinkage, secondary interventions, thrombus localization

## Abstract

**Introduction:**

Endovascular Aortic Repair (EVAR) is widely used to treat abdominal aortic aneurysms. However, long-term success depends on aneurysm sac remodeling, which is influenced by endovascular complications like endoleak. This study investigates the relationship between thrombus localization, IMA diameter and the number of patent lumbar arteries with type 2 endoleak and sac shrinkage.

**Methods:**

A total of 143 patients who underwent elective EVAR were included in the analysis. Preoperative and postoperative thrombus volume and localization were assessed, IMA diameter and lumbar artery counts were evaluated. Thrombus was categorized as either anterior, posterior, circular, or absent.

**Results:**

Within this 143 patients, a postoperative volume reduction of ≥10% was observed in 44 patients. In 65 patients, the thrombus was circularly located whereas 11 patients had posteriorly localized thrombus formation. Posterior thrombus localization was associated with an approximately 7% reduction in aneurysm sac volume during follow-up. In contrast, circular thrombus localization was associated with less sac shrinkage and a higher number of patent lumbar arteries, which likely impeded effective remodeling. The presence of more than three patent lumbar arteries (*p* < 0.001) were negatively correlated with sac shrinkage, suggesting an increased risk of secondary interventions.

**Conclusion:**

These findings suggest that thrombus localization and lumbar artery count may be associated with sac remodeling outcomes after EVAR. Posterior thrombus localization was associated with more favorable sac shrinkage and fewer patent lumbar arteries, although a direct effect on secondary intervention rates was not demonstrated. These findings suggest that thrombus localization and lumbar artery burden may help identify patients at risk for less favorable sac remodeling after EVAR. Such parameters may support closer surveillance and individualized consideration of preventive strategies in selected cases, but prospective validation is required before treatment recommendations can be made.

## Introduction

Elective infrarenal abdominal aortic aneurysms (iAAA) represent a significant concern in vascular health, predominantly addressed through Endovascular Aneurysm Repair (EVAR). Preferred for being a minimally invasive approach, EVAR is the preferred treatment in approximately 70%–80% of cases, largely due to its ability to reduce recovery time, minimize surgical risks, and deliver effective outcomes when compared to traditional open surgery (1).

Despite the initial benefits presented by EVAR, there are challenges that necessitate ongoing vigilance in patient care. Approximately 30% of patients who undergo EVAR are at risk of secondary interventions within a decade following the initial procedure (2). This underscores the importance of ongoing surveillance and early detection of any potential complications to avert severe outcomes. The measurement of maximum aneurysm diameter is regarded as the gold standard for monitoring EVAR outcomes. This metric is pivotal in evaluating the stability and effectiveness of the aneurysm repair over time.

Type 2 endoleak is the most common type of endoleak, attributed to the retrograde flow from the aortic side branches (ASB) [lumbar arteries, Inferior Mesenteric artery (IMA), Accessory renal arteries, etc.] occurring in 8%–44% of patients. Type II endoleak is often considered relatively benign and resolves spontaneously in a substantial proportion of patients during early follow-up; however, depending on the size and number of patent aortic side branches, it may also impair sac regression.

Previous studies have suggested that an enlarged IMA, lumbar artery—typically defined as >3 mm—is independently associated with higher rates of type 2 endoleak and diminished sac regression due to increased collateral perfusion of the aneurysm sac. Assessing IMA size alongside thrombus position and lumbar artery patency may provide a more comprehensive evaluation of factors contributing to sac pressurization and delayed remodeling. Incorporating IMA diameter alongside with lumbar artery measurements into preoperative planning and postoperative surveillance may enhance risk stratification for negative remodeling and inform early consideration of embolization strategies when appropriate. Preoperative thrombus localization and volume have been shown to serve as a robust predictor of sac regression following endovascular aneurysm repair (EVAR), highlighting its potential to guide preoperative planning and surveillance strategies in previous studies and different groups have analyzed thrombus density before EVAR (3, 4). However, some studies have suggested that two-dimensional measurements may not adequately capture these changes because of the irregular three-dimensional morphology of the aneurysm sac (5–7).

Our objective in this study is to thoroughly evaluate the localization of thrombus formation and its impact on aneurysm sac volume change post-EVAR. Understanding thrombus distribution within the aneurysm is vital, as it can play a significant role in the biological behavior of the aneurysm sac and potentially influence the risk of complications. Additionally, assessing the count and patency of lumbar arteries and IMA diameter post-EVAR provides further insights into aneurysm dynamics and potential risks.

Through this research, we aim to improve the accuracy of post-EVAR surveillance strategies. By incorporating thorough assessments of thrombus localization and volumetric changes, we seek to enhance long-term patient prognosis. The ultimate goal is to ensure that patients who undergo EVAR benefit from optimized follow-up protocols that prevent severe outcomes, enhance the durability of the repair, and contribute to improved overall vascular health management.

## Material and method

Our study was conducted retrospectively as an observational single-center study, including 143 patients who underwent elective Endovascular Aneurysm Repair (EVAR) for infrarenal abdominal aortic aneurysms between 2019 and 2023. Out of the 175 patients evaluated, 32 were excluded due to missing preoperative or postoperative computed tomography and follow-up data. Ethical approval for the study was obtained from the Institutional Ethics Committee Review Board (Approval No: 1-25-940, and informed consent was acquired from all participants.

Thrombus localization and the number of patent lumbar arteries were the primary focus of our investigation. Preoperative and postoperative computed tomographic angiography (CTA) with a slice thickness of 0.625 mm was utilized to ensure detailed imaging. Patients admitted urgently with ruptured aneurysms or those lacking control CTA and graft measurement data were excluded. All EVAR procedures were performed by the same experienced cardiovascular surgical team.

Data were systematically categorized into preoperative, intraoperative, postoperative, and follow-up periods. The primary data collected included demographic information, aneurysm diameter, thrombus localization, and thrombus density preoperatively. Graft measurements, procedure duration, fluoroscopy time, and contrast volume for intraoperative parameters and ICU and hospital stay durations, complications, and follow-up CTA measurements postoperatively.

Thrombus volume was assessed using three-dimensional imaging software, quantifying total aneurysm and thrombus volume in cubic centimeters. Patent lumbar artery counts were determined based on CTA imaging, focusing on their role in aneurysm sac pressurization.

Preoperative and postoperative morphovolumetric measurements were performed using the Sarus Workstation program by a cardiothoracic surgeon and a radiologist. Morphovolumetric measurements were performed independently by the two observers, who were blinded to clinical outcomes and postoperative endoleak status during image analysis. A formal interobserver variability analysis was not performed.

Aneurysm volume and thrombus density were measured in Hounsfield Units (HU) across proximal, distal, and middle regions, with averages calculated for further analysis. These measurements were then processed using imaging software Advantage Workstation 4.2 (GE Healthcare Technologies) and recorded in an Excel database.

The number of patent lumbar arteries was specifically analyzed as a critical parameter affecting thrombus pressurization and sac remodeling. Preoperative imaging identified reference points for volumetric measurements, including infrarenal diameter (Diameter A), pre-sac diameter (Diameter B), aneurysm neck length and maximum diameter. Thrombus volume was calculated by subtracting patent lumen volume from the total aortic volume, with this methodology consistently applied to all preoperative and postoperative CTA measurements.

Key endpoints of the study included evaluating the influence of thrombus localization and patent lumbar artery count on aneurysm sac remodeling post-EVAR, alongside their correlation with endoleaks and secondary interventions. The primary endpoint was positive aneurysm sac remodeling, defined as a ≥10% reduction in aneurysm sac volume at follow-up compared with baseline. This threshold was selected as a morphovolumetric marker of favorable sac behavior based on prior EVAR volumetric studies and was intended to represent imaging-defined remodeling rather than a standalone clinical outcome. Secondary endpoints included type II endoleak, secondary intervention, and overall survival/mortality. Diameter-based change (≥5 mm reduction in maximum sac diameter) was analyzed as a secondary supportive metric. Operational success was defined as the absence of conversion to open surgery, intraoperative mortality, Type 1 or Type 3 endoleaks, stent migration, or occlusion detected during complementary angiography. These metrics provided a robust framework for understanding the impact of thrombus-related parameters on patient outcomes.

## Statistical analysis

All statistical analyses were conducted using SPSS statistical software (SPSS for Windows 15.0, Inc., Chicago, IL, USA). The power of the study was calculated using G*Power 3.1.9.7 (Franz Faul, Universitat Kiel, Germany), with a power of 98% achieved based on the sample size and effect size estimates. Normality of continuous variables was assessed using the Kolmogorov–Smirnov test. Continuous variables conforming to normal distribution were expressed as mean ± standard deviation (SD), while non-normally distributed variables were presented as median with interquartile ranges (IQR). Categorical variables were described as frequencies and percentages.

Comparative analyses were performed using independent samples t-tests or Mann–Whitney U tests for continuous variables and chi-square tests or Fisher's exact tests for categorical variables. For paired continuous data, the Wilcoxon signed-rank test was used. Patients were categorized into two groups based on the presence or absence of positive aortic remodeling, defined as a ≥10% reduction in aneurysm sac volume during follow-up.

Multivariable logistic regression analysis was conducted to identify independent predictors of positive remodeling, with thrombus localization and patent lumbar artery count included as key variables. Variables with *p*-values <0.1 in univariate analysis were included in the multivariate model. Odds ratios (OR) with 95% confidence intervals (CI) were calculated for significant predictors. Because the number of positive remodeling events was limited, the final multivariable model was intentionally restricted to a small number of clinically relevant covariates selected from univariate analysis in order to reduce overfitting and preserve model stability. The multivariable findings should therefore be interpreted as exploratory.

The relationship between sac volume reduction and diameter reduction was analyzed using linear regression. Receiver Operating Characteristic (ROC) analysis was used to assess the sensitivity and specificity of thrombus localization and patent lumbar artery count as predictors of sac remodeling and Type 1A endoleak occurrence. Kaplan–Meier survival analysis was performed to evaluate long-term outcomes, stratified by positive remodeling status. A *p*-value of <0.05 was considered statistically significant throughout all analyses.

## Results

Technical success was achieved in 100% of cases, defined as the absence of conversion to open surgery, intraoperative mortality, type I or III endoleak on completion angiography, stent migration, or graft occlusion. Among the 143 patients monitored,132 patients (93.1%) were male, with an average age of 69.08 ± 7.51 years. Hypertension was present in 71.8%, coronary artery disease in 48.1%, and 53.4% had a smoking history. Additionally, 5.3% had a history of cancer. General anesthesia was administered to 84.5%. The mean fluoroscopy time was 10.4 ± 7.5 min, and the average contrast volume used was 42.2 ± 13.7 cm^3^. The average duration in the ICU was 7.1 ± 12.8 h, and the mean hospital stay was 2.9 ± 2.8 days. The follow-up period averaged 25.6 ± 15.02 months.

The preoperative mean aneurysm diameter was 65.5 ± 13.3 mm (range: 54−118 mm). Diameter A was 23.4 ± 3.5 mm, Diameter B was 24.75 ± 4.4 mm, and the aneurysm neck length was 28.7 ± 13.1 mm. The average alpha angle was 39.04 ± 25.25 degrees. The total aneurysm sac volume was 221.7 ± 146.4 cm^3^, and the thrombus volume averaged 104.6 ± 88.6 cm^3^. The mean preoperative thrombus-to-aneurysm volume ratio was 44.5% ± 22.3%. Thrombus density averaged 35.7 ± 13 HU (Table 1). Thrombus localization was circular in 49.6% and posterior in 7.6% of total patients. The mean diameter of the inferior mesenteric artery was 2.2 ± 1.1 mm. 81 out of 143 patients (57%) had 4 or more patent lumbar arteries, while 62 patients (43%) had 3 or fewer patent lumbar arteries.

**Table 1:** Change in preoperative and postoperative morphological measurements.

Parameter	Preoperative (mean ± sd)	Postoperative (mean ± sd)	*P* value
Aneurysm diameter (mm)	65 ± 13	62 ± 16	<0.001
Diameter A (infrarenal diameter mm)	23 ± 3	26 ± 4	<0.001
Diameter B (diameter before sac mm)	24 ± 4	24 ± 4	<0.001
Aneurysm Neck Length(mm)	29 ± 13	30 ± 13	*P* = 0.021
Alpha angle(degrees)	39 ± 25	32 ± 23	<0.001
Aneurysm volume (cm3)	221 ± 146	215 ± 162	*P* = 0.487
Thrombus volume (cm3)	104 ± 88	159 ± 145	<0.001
Thrombus density (HU)	35 ± 13	34 ± 11	*P* = 0.177
Thrombus/Aortic volume %	44 ± 22	65 ± 16	<0.001

SD: Standard deviation.

A total of 83.2% of patients were on beta-blockers or antihypertensive treatments. Statin use was observed in 42%, and metformin in 10.7%. Approximately 60.3% were on aspirin, and dual antiplatelet therapy was used by 32.8%.

During follow-up, 33 patients (23%) had endoleaks: 6 patients with type 1A, 7 with type 1B, 14 with type 2, and 6 with type 3. No early mortality was observed. Late mortality was observed in 21 patients (14%), with cardiac symptoms being the primary cause (38%). Mortality due to aneurysms rupture was 1.5% in patients who refused treatment for type 3 endoleaks. Secondary interventions were performed in 17 patients (11.8%), and a total of 19 patients underwent additional procedures. Iliac occlusion was detected in 2 patients, one underwent femorofemoral crossover bypass, and the other underwent femoral embolectomy with iliac extension.

**Figure 1:**
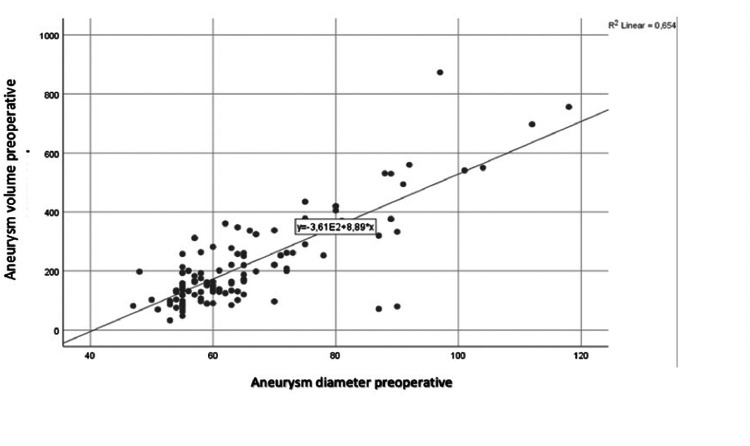
Correlation between preoperative aneurysm diameter and preoperative anuerysm volume.

Postoperative morphological changes in comparison to preoperative measurements are presented in Table 1. Postoperatively, while there was a significant reduction in aneurysm diameter, no significant decrease in aneurysm volume was noted. Both Diameter A and B showed significant increases (*p* < 0.001), while the alpha angle significantly decreased (*p* < 0.001). A positive correlation was observed between preoperative aneurysm diameter and preoperative aneurysm volume (Figure 1)

**Figure 2:**
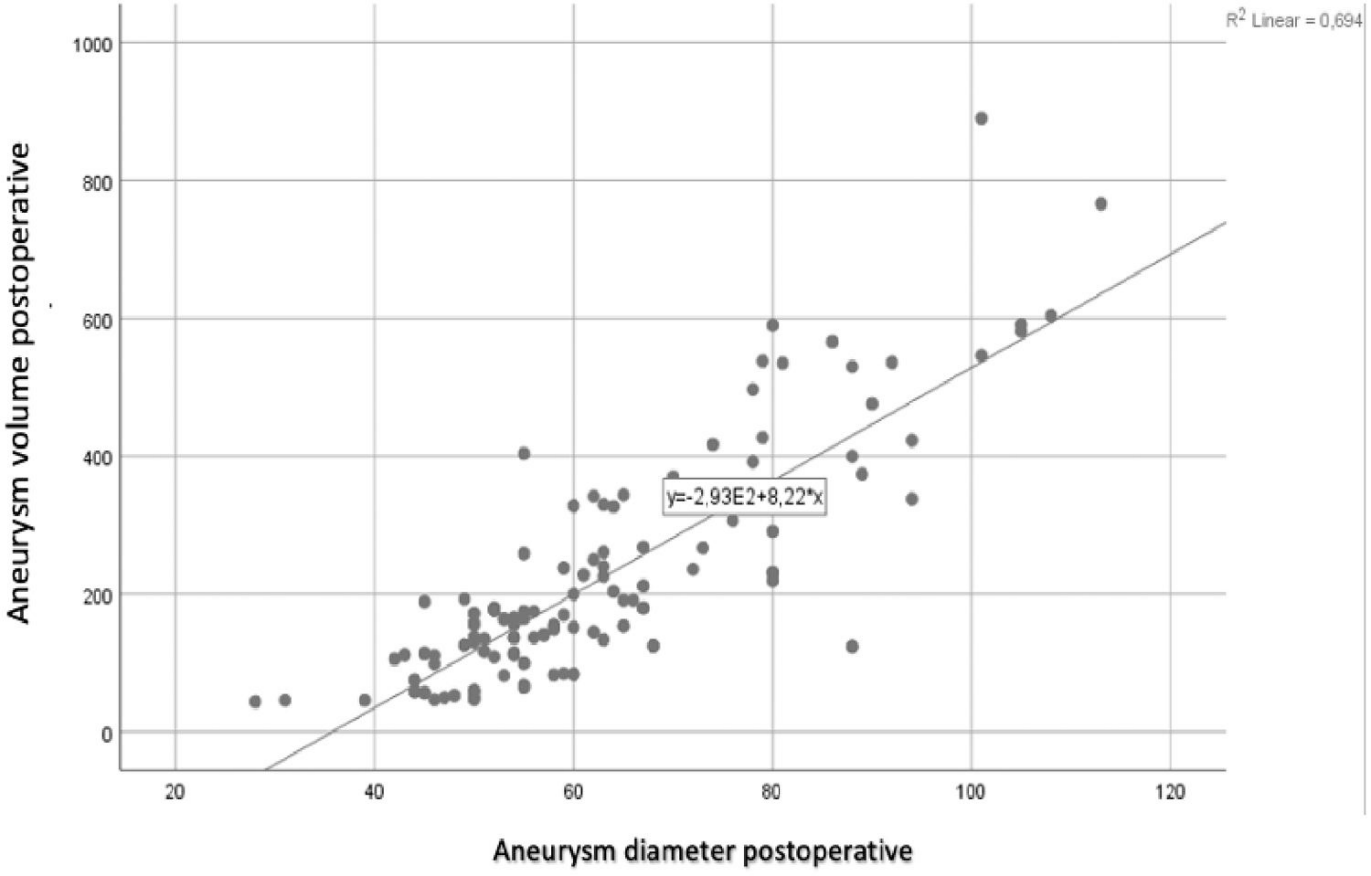
Preoperative and postoperative diameter-volume correlation.

A strong correlation was found between preoperative and postoperative aneurysm diameter and volume, with correlation coefficients of r = 0.809 and r = 0.833, respectively (Figure 2).

A 10% or more volume decrease was classified as positive remodeling. Based on this, 44 patients (30%) exhibited positive remodeling. Additionally, 50 patients (34.9%) exhibited a ≥5 mm reduction in maximum sac diameter, which was analyzed as a secondary supportive remodeling metric complementary to volumetric assessment. We categorized thrombus localization into four distinct groups: no thrombus, anterior, posterior, and circular. This categorization allowed us to analyze how thrombus position within the aneurysm sac affects sac behavior and remodeling. The data revealed that patients with posteriorly located thrombus experienced significantly fewer patent lumbar arteries, averaging 3.1 ± 1.6. This contrasts with other thrombus locations and was statistically significant (*p* = 0.002) (Table 2).

**Table 2:** Thrombus localization, volume, number of lumbar arteries and comparision of other parameters .

Parameter		Thrombus Localization				P*
		None1(*n* = 9)	Anterior2(*n* = 39)	Posterior3(*n* = 30)	Circular4(*n* = 65)	
Aneurysm Diameter (mm)	Preop	67.8 ± 9.2	62.9 ± 10.6	69.9 ± 18.4	65.2 ± 12.7	0.250
	Postop	67.2 ± 11.7	60.1 ± 15.3	67.3 ± 19.0	60.5 ± 16.3	0.271
	*Δ*Pre-Post	−0.7 ± 14.7	−2.8 ± 8.2	−2.6 ± 9.5	−4.6 ± 9.1	0.577
Volume (cm^3^)	Preop	221.8 ± 73.8	193.1 ± 155.3	263.6 ± 180.7	223.3 ± 131.3	0.351
	Postop	257.2 ± 149.8	212.4 ± 173.8	234.6 ± 195.0	206.6 ± 146.0	0.824
	Remodeling %	12.1 ± 35.8	10.9 ± 40.9	−7.0 ± 42.2	8.2 ± 108.0	0.017
Number of Lumbar Arteries		5.5 ± 1.8	4.3 ± 1.7	3.1 ± 1.6	3.6 ± 1.6	0.002
Ima Diameter(mm)		2.5 ± 0.8	2.3 ± 1.2	2.6 ± 1.0	2.0 ± 1.2	0.197
Alpha Angle(^o^)	Preop	71.2 ± 26.9	35.6 ± 22.6	46.0 ± 21.9	35.6 ± 25.6	0.003
	Postop	54.7 ± 26.9	27.0 ± 18.8	38.3 ± 22.4	29.9 ± 24.4	0.021
**Type 2 endoleak (*n*** **=** **14) (%9.79)**		**None (*n*** **=** **0) %0.0**	**Anterior(*n*** **=** **5) %3.49**	**Posterior(*n*** **=** **2) %1.39**	**Circular(*n*** **=** **7) %4.89**	**0**.**21**

A notable finding was the association of posterior thrombus localization with sac volume reduction. A statistically significant decrease in aneurysm sac volume of approximately 7% was observed exclusively in patients with posterior thrombus localization (*p* = 0.017).

In addition to that our analysis demonstrated that patients with three or fewer patent lumbar arteries were more likely to experience positive remodeling, defined as a ≥10% reduction in aneurysm sac volume. This was supported by a *p*-value of <0.001, indicating a statistically significant association.

Further analysis using multivariate logistic regression confirmed this relationship. Having ≤3 patent lumbar arteries was identified as an independent predictor for positive remodeling, with an odds ratio (OR) of 3.80 (95% CI: 1.56−9.22; *p* = 0.003).

Comparative analysis showed significant differences in statin use (*p* = 0.04) and dual antiplatelet therapy (*p* = 0.035) between patients with and without positive remodeling. Additionally, thrombus presence in the neck (≥25%, *p* = 0.049), ≤3 patent lumbar arteries (*p* < 0.001), and endoleak presence during follow-up (*p* = 0.003) were significant in univariate analysis, whereas IMA diameter showed a non-significant trend (*p* = 0.071) (Table 3).

**Table 3:** Endoleak types and thrombus localization with Ima diameter and number of lumbar arteries.

Parameter		Endoleak Type *N* = 33				P*
		1A	1B	2	3	
		*n* = 6	*n* = 7	*n* = 14	*n* = 6	
**Number of Lumbar Arteries**		5,5 (2,0–8,0)	4,0 (1,0–7,0)	4,0 (2,0–6,0)	3,5 (3,0–6,0)	0,757
**Ima Diameter(mm)**		2,8 (1,7–3,1)	2,8 (0,0–5,2)	2,4 (0,0–4,2)	2,8 (1,7–3,3)	0,984
**Thrombus Localization**	None	2 (%1.3)	2 (%1.3)	0 (%0,0)	0 (%0,0)	–
	Anterior	1 (%0.6)	2 (%1.3)	5 (%3.49)	3 (%2.09)	
	Posterior	0 (%0,0)	2 (%1.3)	2 (%1.3)	0 (%0,0)	
	Circular	3 (%2.09)	1 (%0.6)	7 (%4.89)	3 (%2.09)	

The final multivariable logistic regression model included four covariates selected from the univariate analysis (*p* < 0.10): dual antiplatelet therapy use, number of patent lumbar arteries, endoleak presence during follow-up, and thrombus presence in the aneurysm neck (≥25%). Given the limited number of remodeling events, this model was deliberately simplified to reduce overfitting and should be interpreted cautiously.

Multivariate logistic regression showed that the absence of dual antiplatelet therapy had an OR of 0.3 (95% CI: 0.11–0.81; *p* = 0.017), ≤3 patent lumbar arteries had an OR of 3.80 (95% CI: 1.56–9.22; *p* = 0.003), and absence of endoleak had an OR of 6.16 (95% CI: 1.62–23.41; *p* = 0.008) as independent predictors for positive remodeling. ROC analysis indicated an alpha angle >47.5 degrees was associated with Type 1A endoleak, with 75% sensitivity and 69.7% specificity.

Preoperative aneurysm volumes >233.5 cm^3^ were linked to secondary intervention, with 80% sensitivity and 76.7% specificity. Similarly, preoperative thrombus volumes >204 cm^3^ were linked to secondary intervention, with 73.3% sensitivity and 81.6% specificity. Nevertheless, no association was found between preoperative thrombus volume (*p* = 0.537) and the ratio of thrombus volume to aortic volume (*p* = 0.164) with endoleaks, regardless of endoleak type (Table 4).

**Table 4:** Comparisons by endoleak status.

Parameter	Endoleak		*P*
	None (*n* = 110)	Yes (*n* = 33)	
TV Preop	107,4 ± 87,8	96,3 ± 91,9	0,537^a^
TR/AO %	46,1 ± 21,3	39,8 ± 24,8	0,164^a^

a Independent Samples t Test (Mean ± SD).

TV preop, preoperative thrombus volume.

TR/AO %: Percentage of preoperative thrombus volume to aortic volume.

It was also found that having 4 or more lumbar arteries and the diameter of the IMA were not significantly associated with type 2 endoleak (Table 5).

**Table 5:** Relationship between IMA diameter and patent lumbar artery count and type 2 endoleak.

Parameter	Type 2 ENDOLEAK	OTHER	*P* value
İMA D.(mm)	2.4 ± 1.2	2.2 ± 1.1	0.413
≥4 LOMBER ARTERY	11patient (% 78.6)	70 patient (%53)	0.088

D, Diameter.

**Figure 3:**
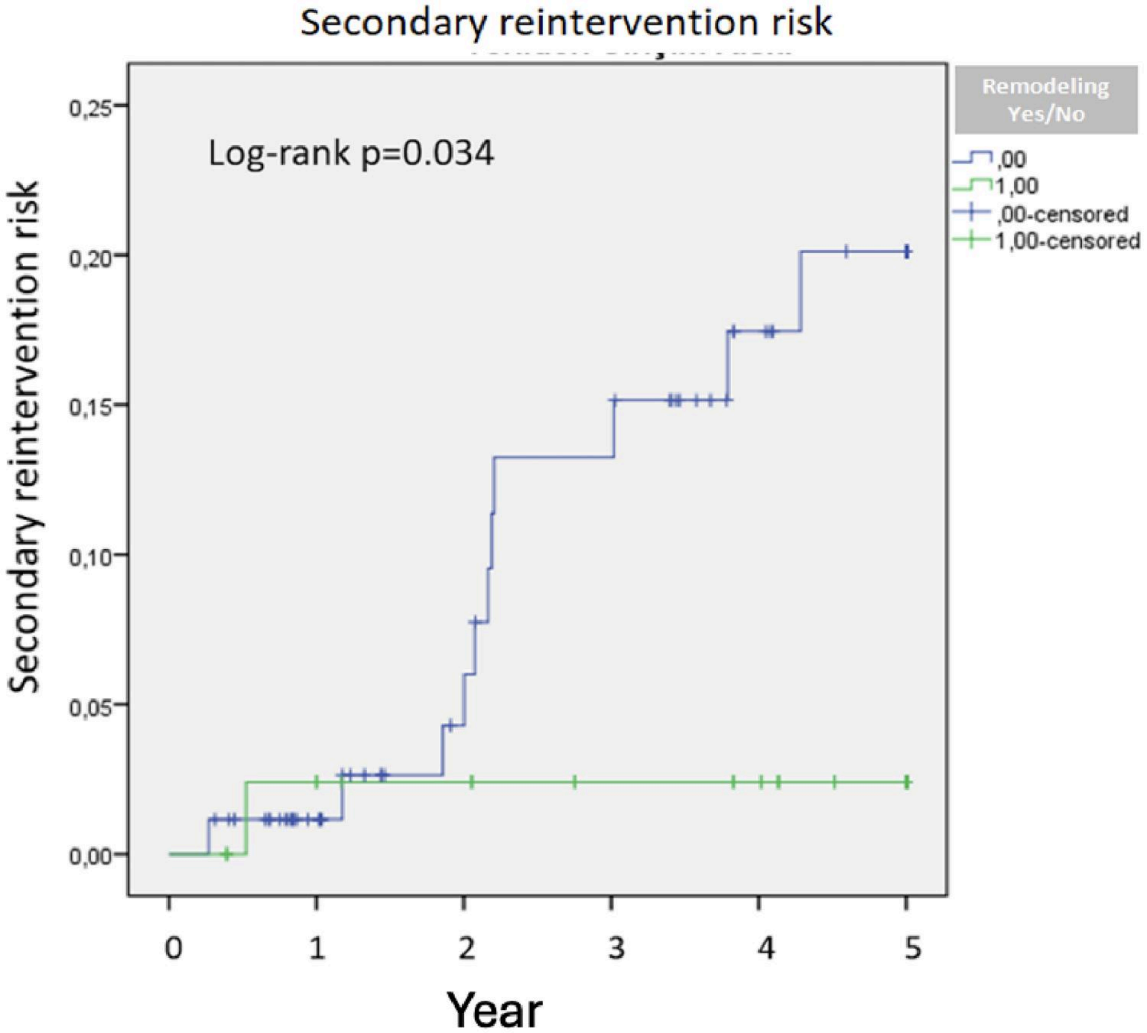
Secondary reintervention risk.

Patients without remodeling after two years were at increased risk of reintervention. At baseline, 99 patients were classified as having no sac remodeling and 44 patients as having positive sac remodeling. During follow-up, the number of patients remaining under observation gradually decreased due to censoring events and varying follow-up duration. The Kaplan–Meier analysis demonstrated a significantly higher risk of secondary intervention in patients without sac remodeling (log-rank *p* = 0.034) (Figure 3). No significant survival difference was observed between those with and without positive remodeling in Kaplan–Meier analysis; however, given the mean follow-up duration, longer-term estimates should be interpreted cautiously (*p* = 0.88).

## Discussion

Our study highlighted the potential role of thrombus localization, IMA diameter, and patent lumbar arteries in sac remodeling after EVAR. Thrombus localization, an important determinant of sac dynamics, was associated with the need for secondary interventions (*p* = 0.034).

In the contemporary era, more than 70% of infrarenal abdominal aortic aneurysms are treated with endovascular aneurysm repair (EVAR) (1). Despite the minimally invasive nature of EVAR, it faces persistent criticism due to risks such as ongoing rupture, the need for re-intervention, continuous surveillance requirements, and associated costs (2).

The location of the thrombus within the aneurysm sac played an important role in determining postoperative remodeling. Posterior thrombus location was associated with the most significant sac reduction and the lowest number of patent lumbar arteries (*p* = 0.017). However, this finding should be interpreted cautiously because the posterior thrombus subgroup was small, and the result should therefore be considered hypothesis-generating rather than definitive. One possible explanation is that posterior thrombus localization may facilitate thrombosis or partial coverage of lumbar artery orifices, thereby reducing retrograde collateral flow and favoring sac shrinkage. Recent contemporary evidence also supports that post-EVAR sac behavior and type II endoleak occurrence are influenced by anatomical and morphometric parameters (8).

From a hemodynamic perspective, the distribution of intraluminal thrombus (ILT) within the aneurysm sac may influence intra-sac pressure transmission and wall stress patterns. Experimental and computational studies have suggested that ILT can partially attenuate pulsatile pressure transmission from the aortic lumen to the aneurysm wall, thereby modifying local wall stress (9, 10). When thrombus is predominantly located posteriorly, it may mechanically cover or compress the orifices of lumbar arteries, potentially reducing retrograde collateral perfusion into the aneurysm sac. This configuration may facilitate earlier sac depressurization and may contribute to progressive sac shrinkage following EVAR. Conversely, circumferential or anterior thrombus distribution may allow persistent collateral inflow from lumbar branches, maintaining residual sac pressurization and limiting aneurysm regression. These hemodynamic mechanisms may partly explain the differences in sac remodeling observed in our cohort.

In contrast, circumferential thrombus location involving the anterior, posterior, and lateral walls was associated with impaired remodeling. This finding emphasizes the importance of considering both thrombus volume and location during preoperative planning and postoperative surveillance.

Our findings align with previous studies that identified large preoperative thrombus volume as a significant predictor of less favorable sac shrinkage, underscoring the necessity of volumetric analysis in postoperative follow-up protocols. Patients with preoperative thrombus volumes exceeding 204 cm^3^ had a significantly higher rate of secondary interventions (*p* = 0.002). As suggested in the literature, larger thrombus volumes may be associated with increased intrasac pressure, which could contribute to impaired sac shrinkage. This aligns with Yeung et al., who reported that substantial thrombus burdens reduced the likelihood of sac shrinkage (3). Preoperative thrombus volume was not statistically associated with endoleaks (*p* = 0.537); however, our study highlighted that thrombus localization influenced sac shrinkage and affected sac remodeling by impacting the number of patent lumbar arteries. The significance of sac morphology will become more apparent in studies involving larger patient groups or those with type 2 endoleaks. Conversely, lower thrombus volumes were associated also improved patient outcomes without secondary intervention requirement. Li et al. suggested that smaller thrombus volumes, in conjunction with fewer patent lumbar arteries, resulted in reduced incidence of Type 2 endoleaks, which are predominantly driven by retrograde flow from these collateral vessels (11–16). Our findings reinforce this observation, as patients with ≤3 patent lumbar arteries were significantly more likely to achieve positive remodeling (*p* = 0.003). Notably, positive remodeling was observed in 44 patients (30%), with a strong correlation between sac volume reduction and improved outcomes. We acknowledge that the ≥10% volume reduction threshold should be interpreted as an imaging-based remodeling definition rather than a direct surrogate for clinical success, but it provides a standardized volumetric framework for comparing postoperative sac behavior. These results align with Franchin et al., who highlighted enhanced sac shrinkage in cases with reduced thrombus volumes and diminished collateral flow (12).

Preoperative sac volumes exceeding 233.5 cm^3^ were identified as significant predictors of secondary interventions (*p* = 0.001), further emphasizing the value of volumetric thresholds in risk stratification. Larger aneurysms, as indicated by both sac and thrombus volumes, were more prone to complications. Montelione et al. corroborated these findings, demonstrating the utility of volumetric analysis in tailoring treatment strategies for larger aneurysms (13).

The presence of endoleaks during follow-up was another critical determinant of remodeling outcomes (14). In our study, no association was found between thrombus localization (*p* = 0.21) or thrombus volume (*p* = 0.47) and type 2 endoleak. Although several previous studies have reported a relationship between collateral anatomy—particularly inferior mesenteric artery diameter and the number of patent lumbar arteries—and the occurrence of type II endoleak, our cohort did not demonstrate statistically significant associations. Accordingly, our conclusions were revised to focus on sac remodeling, which was the principal outcome directly supported by our data.

Other anatomical factors, besides thrombus localization such as dual antiplatelet therapy (DAPT) use, may influence the development of type 2 endoleak. In our clinical practice, DAPT is the first pharmacological modality we discontinue when an endoleak is detected**,** particularly in cases of type 2 endoleak. Antiplatelet agents can impede intrasac thrombosis by reducing platelet aggregation, which may contribute to the persistence of retrograde collateral flow. Therefore, early cessation of DAPT is considered an essential step to promote spontaneous thrombosis within the aneurysm sac and facilitate sac regression before proceeding to more invasive secondary interventions. Moreover Bargelli et al., who reported that the absence of volume reduction at six months strongly predicts endoleak persistance independent of type (17). These results reinforce the need for prompt identification and management of endoleaks along with ulderlying causes to mitigate long-term complications as also reported in another study (18–20).

Along with the use of dual antiplatelet therapy (DAPT), statins also significantly influenced remodeling outcomes. While the absence of DAPT was associated with lower odds of positive remodeling (OR 0.3, 95% CI 0.11–0.81; *p* = 0.017). Statin use, known for its pleiotropic effects, including anti-inflammatory and protease-inhibitory properties, was linked to improved sac regression. However, only 54% of patients with positive remodeling in our cohort were on statins, highlighting an area for optimization in clinical practice. These findings are consistent with Raux et al., who reported that statin therapy was associated with sac shrinkage and reduced cardiovascular mortality after EVAR (21–23).

Although collateral perfusion through lumbar arteries and the IMA provides a plausible hemodynamic explanation for impaired sac remodeling after EVAR, our cohort did not demonstrate a statistically significant association between thrombus-related parameters and type II endoleak. Therefore, the present study should primarily be interpreted as an analysis of sac remodeling rather than of type II endoleak prediction. The discussion of collateral-flow mechanisms is presented as biological context for sac behavior rather than as a direct finding established by our results. While the literature remains heterogeneous regarding routine pre-emptive embolization, several studies suggest that selective embolization of the IMA or lumbar arteries may reduce the likelihood of persistent type 2 endoleaks and facilitate early sac regression. This approach could be used as a center-specific principle in anatomically high-risk cases to optimize sac remodeling and reduce the need for secondary interventions (24, 25). Ide et al. concluded that impact of patent LA on sac enlargement was significant with the presence of a patent IMA (26). Chew et al. suggested preemptive embolization of the IMA and LAs reducing secondary intervention for sac growth (27). Kondov et al. found that patients with IMA diameter ≥3 mm and more than 3 patent LAs carry a higher risk of developing type 2 endoleak after EVAR (28). Besides Mulay et al. concluded no differences in overall survival between type 2 endoleak presence and absence. Patients who had secondary intervention did not have better survival. In light of these heterogeneous results, Type 2 endoleak management differs among centers (29).

Our findings underscore the necessity of integrating thrombus localization and patent lumbar artery assessments into EVAR planning and surveillance protocols. These parameters, combined with volumetric analysis, provide a comprehensive understanding of aneurysm behavior, enabling earlier detection of complications and more tailored interventions. This approach represents a significant step forward in optimizing patient outcomes and minimizing the need for costly and invasive secondary procedures.

## Limitations

Several limitations of our study should be acknowledged. The relatively small sample size and retrospective design may limit the generalizability of our findings. In addition, formal interobserver variability for thrombus localization and morphovolumetric measurements was not assessed, which is an important limitation for an imaging-based analysis. Additionally, the overlap of the follow-up period with the COVID-19 pandemic disrupted routine imaging schedules, potentially introducing bias. Finally, variability in device selection and imaging protocols highlights the need for standardized approaches in future research. Larger, multicenter studies with consistent methodologies are essential to validate our findings and refine EVAR surveillance strategies.

Only a small proportion of patients exhibited posterior thrombus localization (7.6%), meaning that one of the manuscript's principal observations is based on a limited subgroup. This restricts statistical robustness, increases uncertainty in subgroup comparisons, and requires confirmation in larger multicenter cohorts. Also since multivariable analysis was performed using a restricted set of covariates, the number of outcome events remained relatively limited for regression modeling, and the adjusted estimates should be interpreted with caution.

Finally, the mean follow-up duration limits the reliability of longer-term survival estimates derived from Kaplan–Meier analysis.

## Conclusion

Our study suggests that thrombus localization and patent lumbar artery count may be associated with sac remodeling after EVAR. Posterior thrombus localization and fewer patent lumbar arteries were associated with more favorable sac behavior, whereas non-posterior thrombus distribution and greater lumbar artery patency were associated with less favorable remodeling. These findings may improve postoperative risk stratification and help identify patients who may benefit from closer surveillance. However, because of the retrospective design and limited subgroup size, these results should be considered hypothesis-generating and require confirmation in larger prospective studies before guiding treatment selection.

## Data Availability

The original contributions presented in the study are included in the article/Supplementary Material, further inquiries can be directed to the corresponding author.
